# The caregiving perspective in heart failure: a population based study

**DOI:** 10.1186/1472-6963-13-342

**Published:** 2013-09-03

**Authors:** Patricia M Davidson, Amy P Abernethy, Phillip J Newton, Katherine Clark, David C Currow

**Affiliations:** 1 Centre for Cardiovascular and Chronic Care, Faculty of Health, University of Technology Sydney, PO Box 123, Level 7, Building 10 Jones Street, Broadway, Sydney, NSW 2007, Australia; 2 Division of Medicine Oncology, Duke University Medical Center, 3436, Durham, NC 27710, USA; 3 Director & Area Director of Palliative Care, Calvary Mater Newcastle, University of Newcastle, Waratah, Newcastle, NSW 2298, Australia; 4 Professor, Discipline of Palliative and Supportive Services, Flinders University Flinders Centre for Clinical Change, Bedford Park, 5042, South Australia, Adelaide, Australia; 5 School of Nursing, Johns Hopkins University, 525 N. Wolfe Street, Baltimore, MD 21205, USA

**Keywords:** Heart failure, Caregivers, Australia

## Abstract

**Background:**

Heart failure (HF) is a frequent condition in the elderly and mortality is high. This study sought to describe the profile of those providing care in the community and their needs.

**Methods:**

The South Australian Health Omnibus is an annual, random, face-to-face, cross sectional survey conducted within the state. Having standardized data to the whole population, the study describes the subset of the population who identify that they actively cared for someone at the end of life with HF in the five years before survey administration.

**Results:**

Three hundred and seventy three respondents (2.0% of the whole population; 4.9% of caregivers) reported being a caregiver of someone with HF. There were 84 active caregivers (day-to-day or intermittent hands on caregivers) for people with HF. Mean age for caregivers for those with HF was much higher than other caregivers (55.7 *vs* 49.4; *p* < 0.001) with care lasting for an average of 48.9 months (SD 66.2). People caring for those with HF were far less likely to access specialist palliative care services (38.1% *vs* 60.9%; *p* < 0.0001) despite having much greater levels of unmet needs for physical care 28.3% *vs* 14.1%; *p* = 0.008).

**Conclusion:**

Study findings suggest that there is a significant burden placed on caregivers for people with HF over extended periods in the community. There are differences in access to services for these caregivers compared to those dying from other conditions, particularly cancer.

## Background

Heart failure (HF) is a chronic illness with a poor prognosis [1,2]. In the elderly, caregiving needs are often accentuated and support systems are not always as readily accessible [3]. Increasingly there is interest in developing models of care to support individuals and their families who face dying with HF [1,4,5]. The impact of HF on individuals and health care systems are well described, yet much less is known about the impact of caregiving on informal caregivers, who are commonly family members [6,7]. The physical, psychological and social demands of caregiving can impact adversely on the well-being of caregivers [8]. Hwang and colleagues have noted that care provided for individuals with HF is much more extensive than that provided with usual spousal assistance and support [9].

The increasing emphasis on community based HF management is placing additional pressure on family caregivers [10]. As a consequence, there is an increased stress on carers to provide physical care as well as social and emotional support. As in many other chronic conditions, caregiving has benefits and burdens: on the one hand it represents an opportunity for increased intimacy and connection, yet is also associated with significant burden, constancy of role, poor health outcomes for the caregiver and distress [6]. This is particularly the case if the carer is a spouse or in poor health themselves [11]. Consequently, there is a need to document and explore the role of caregiving in HF in the community [12]. Although there are some universally shared characteristics of caregiving, there are potentially some discrete differences among diagnostic groups of the care recipient. Although the merit of focussing on variable illness trajectories has been challenged [13], there is merit in understanding the extent of unmet needs and comparison of health service utilisation between different caregivers for different diagnoses.

Although there has been extensive research on caregivers in many other clinical setting, research is limited in CHF and there are sparse recommendations to address unmet needs for support [14]. Available data suggests that caregiver burden increases if the caregiver experiences poor mental and/or physical health and has limited social and professional support [15]. Patients who are enrolled in specialty disease management programs are likely to have a level of caregiver support whereas those in primary care settings are less likely to have this support underscoring the need to undertake a population based approach to assessing needs.

Current guidelines recommendations suggest that interventions to reduce caregiver burden should focus on improving social support as well as control over their situation [16-18]. A comparison of caregivers with cancer, chronic obstructive pulmonary disease and HF demonstrated that caregiver resources not patient diagnosis or illness severity were associated with caregiver burden [19].

The majority of the data involving caregiver is focussed in the hospital and the immediate post discharge period [20], little is known about informal community caregiving in HF, particularly in the period leading to death. In Australia, the average time from referral to a palliative care service to death is 102 days, with 28.5% of people accessing specialist palliative care for longer than six months nationally [21]. In parallel with international trends, the proportion of those people with non-malignant conditions accessing specialist palliative care services is increasing.

This study describes the role of caregivers of people with HF from a population perspective, not simply those who access specialist or tertiary services [21]. This is more likely to generate a real world perspective of the increasing numbers of people providing care in the community.

Using a population-based approach, the aim of this study was to describe the characteristics of caregivers for people with HF at the end of life and compares these with other caregivers [21]. We have previously reported the findings of the survey related to met and unmet needs, [22] short- and long-term needs [23] and the relationship between perceived comfort and accessing palliative care services [22].

## Methods

A random annual population-based health survey, the South Australian Health Omnibus Survey, has been conducted annually since 1991 [24]. An omnibus survey is a method of quantitative research using a stratified sample where data on a wide variety of subjects is collected during the same interview. Multiple researchers contribute individual questions while sharing the common demographic data collected from each respondent [21]. The Omnibus Survey is a face-to-face, cross-sectional survey. The survey is administered by a commercial research organisation with government support. There is a cost to researchers for each question included in the survey. Since 2000, information has been collected about the experiences of respondents who had someone close to them die from a life-limiting illness in the five years before responding [21]. A detailed response of the question route is describe elsewhere [23]. The questions are designed to access information relating to the nature of the respondent’s relationship with the deceased person, the extent of their involvement in caregiving, their perception of needs (met or unmet), the use of palliative care and other services, and the main reason if a palliative care service was not used. For example caregiving was defined for the respondent as follows “Care’ includes attention to any of the needs of the person, including hands-on care, overnight care, respite, shopping, collection of medications, taking to appointments, emotional support, bathing, etc.” To incorporate differing levels of caregiver burden into the analysis, respondents were asked if they provided: “day-to-day hands-on care” (care 5–7 days per week); “intermittent hands-on care” (care 2–4 days per week); or, “rare hands-on care” (care 1 or less days per week). Pilot testing of the questionnaire with 50 members of the general public for comprehension and usability occurred each year and no changes were made to wording as a result of piloting. For the purposes of this study, the carers who identified themselves were compared to the remaining sample of caregivers, the majority of who reported deaths from cancer or end-stage respiratory disease.

### Sampling schema

From September to December (2001–2007 inclusive), more than 4500 properties were approached annually to participate. In metropolitan areas, a starting point was randomly selected for a selection of 341 Australian Bureau of Statistics collector’s district. In non-metropolitan areas, households were selected using 100 starting points state-wide; all towns with a population greater than 10,000 were included and towns above 1000 were randomly included with chance of inclusion proportional to the size of the town. From the randomly selected starting point within each collector’s district, 10 dwellings were selected using a skip pattern of every fourth household in a pre-arranged algorithm. A cluster size of 10 was used at each of the non-metropolitan starting points. One interview per household was conducted with the person over the age of 15 who had most recently had a birthday. If that person declined, no data were collected from that household. Interviews were conducted face-to-face by trained interviewers. Prompt cards were provided for selected answers to allow responses to be categorised. Data were entered using double entry techniques. Any missing responses were followed up by telephone. For quality assurance, 10% of each interviewer’s respondents were randomly selected and recontacted to confirm eligibility and responses. Aggregated data were then de-identified. These processes apply to the whole survey, are unchanged since the survey’s inception in 1991, and could not be modified just for the questions relating to the end of life due to the Omnibus study methodology and the capacity to add limited items to the overall study structure. In spite of this limitation, the capacity to obtain a population based approach to caregiving at the end-of-life for individuals with HF is an important opportunity.

### Setting

Australia supports a system of universal healthcare insurance which may be supplemented by private health insurance. Palliative care services can be accessed concurrently with other specialist services [25].

South Australia (SA) has a population of 1.54 million people [26]. Specialist palliative care services within SA span a range of different service delivery models, from large regional multidisciplinary teams within the capital city to single clinical nurses in small rural locations. Each service covers a geographic region, encompassing public and private hospitals, free-standing palliative care units, outpatient clinics and a community care team working in conjunction with general practitioners and community nurses. Almost 60% of people with a life-limiting illness are referred to specialist palliative care services in SA [21]. There were five metropolitan and 14 rural/regional publicly funded specialist multidisciplinary palliative care services. The primary eligibility criterion to access these services is a life-limiting illness, irrespective of diagnosis or prognosis. These services are comprised of a team of multidisciplinary health professionals whose substantive training and work are within palliative care.

### Statistical analysis

Data were weighted according to the 10 year age group, sex, geographic profile and country of birth using the 2006 standardised population of SA [26]. Descriptive statistics were used for respondent and patient characteristics. Multiple year comparisons were enabled by a weighting factor to ensure standardized populations were maintained [23]. Descriptive statistics were used to summarize respondent characteristics and frequency of responses. Cross tabulations using Chi square included (for the person with the life-limiting illness) principal diagnosis, (and for the caregiver) a range of demographic and caregiving characteristics. An exploratory logistic regression analysis using data from active caregivers was undertaken with factors from the cross tabulations most likely to distinguish caregiver characteristics (age of respondent as a continuous variable, highest level education, palliative care service use, Index of Socioeconomic Disadvantage (SEIFA) index) [27] and each of the unmet need domains (instrumental support, information, emotional support, finances) categories. All of the factors in the regression were biologically plausible. The model used cause of death (heart failure versus other causes) as the dependent variable to examine the caregiving needs specific to heart failure deaths. A *p*-value <0.05 was taken as significant.

PWAS 18.0 (SPSS Corporation Chicago Il) was used for statistical analysis. A sensitivity analysis using data with unweighted data was used to confirm the direction and magnitude of findings.

### Ethics and consent

The Health Omnibus survey received State ethics committee approval in 1991, and ethical review continues annually. Verbal consent was obtained from all participants. In South Australia, informed consent can be given by anyone over the age of 15 and continuing participation in this interview was taken as continuing consent.

## Results

Forty three percent of respondents (7915/18224) (unweighted) had someone close to them die in the last five years from an expected death. Approximately 5% of people who had experienced a death of someone close in the last 5 years (4.9% (*n* = 373; weighted) of all respondents) attributed this cause to HF, of whom 22.5% (84) provided active care (day-to-day or intermittent hands-on care). Active carers for people dying of HF were statistically likely to be more elderly (55.7 years; SD15.1) compared to other active caregivers (49.4 years; SD 16.6; *p* = 0.001). The period of informal caregiving lasted on average for 48.9 ± 66.2 months. Almost half of caregivers for HF described that the experience was worse than they expected or were unaware of what to expect. Eighty percent of HF caregivers felt that they could move on following the death of the person close to them.

Table 1 compares the respondent characteristics, place of death and uptake of palliative care services between active carers of individuals with HF and other conditions, where the cause of death was ‘expected’. People with HF were statistically far less likely to access specialist palliative care services (provided by specialist palliative care providers) and likely to access palliative care services (provided by community and primary care providers) (38.1% compared with 60.9%; *p* < 0.0001). Almost half (45.0%) reported that the person they care for was comfortable at the end of life.

**Table 1:** Cross tabulation – respondents to the South Australian Health Omnibus who had someone close to them die in the 5 years before responding from an ‘expected’ death and provided a level of active hands-on care (day-to-day and intermittent care; n = 1504; weighted data)

**Respondents who had someone close to them die from an expected death in the 5 years before responding who provided care for someone with …**						
	**…heart failure**			**… a diagnosis other than heart failure**		
**Respondents**						
Factors that do not change as caregiving is relinquished						
	**Factor**	**Fraction**	**%**	**Fraction**	**%**	p value**
	Gender – male	30/84	35.7	526/1420	37.0	0.806
	Age of respondent – 65+	23/84	27.4	283/1420	19.9	0.099
	Educational attainment – beyond school	36/84	42.9	764/1420	53.8	0.051
	Country of birth – non-English speaking	12/84	14.3	142/1420	10.0	0.208
	Relationship to the deceased – spouse	14/84	16.7	178/1420	12.5	0.270
Factors that may change as caregiving is relinquished						
	Household income ≤ AU$60,000	50/68	73.5	819/1240	66.0	0.203
	Current work status – full or part time	31/74	41.9	668/1205	55.4	0.023
	Region of residence – metropolitan	52/83	62.7	952/1420	67.0	0.409
	SEIFA index –lowest 60%	47/84	56.0	626/1420	44.1	0.034
Caregiving characteristics						
	Level of care – day-to-day	35/84	41.7	634/1420	44.6	0.593
	Length of care – ≤ 1 year	30/51	58.8	540/969	55.7	0.664
	Had enough support	19/73	26.0	339/1207	28.1	0.704
Post-care factors						
	Time since death. ≤ 2 years	48/83	57.8	803/1412	56.9	0.863
	Moving on with life – able to move on	66/83	79.5	1133/1408	80.5	0.832
	Would care again – yes	21/23	91.3	279/299	93.3	0.713
	Sought help for grief or wished they had – yes*	11/30	36.7	226/590	38.3	0.857
**The deceased**						
	Age of the deceased >65*	26/30	86.7	428/592	72.3	0.094#
	Comfortable or very comfortable in the last 2 weeks of life*	9/20	45.0	156/378	41.3	0.741
	Place of death – institution (hospital or hospice)*	20/29	69.0	405/593	68.3	0.940
**Service factors**						
	Palliative care service use – yes	32/84	38.1	865/1420	60.9	0.000

* only asked in 2006.

# Fisher’s exact test.

** Using a Bonferroni correction, a significant p value should be set at 0.0025: Fisher’s Exact test used.

Assistance with specific aspects of care was explored with active caregivers. The only difference seen between those caring for someone with HF and those with other life-limiting illnesses was in the unmet needs for hope with physical care which was twice as frequent in people with HF (28.3% *vs* 14.1%; *p* = 0.008).

Table 2 reports the unmet needs identified in aggregated domains by respondents responding to two questions in 2006–2007 (*n* = 5547) who were active caregivers for someone at the end of life at some time in the five years before responding and knew whether or not they would provide care again (*n* = 73). The need for additional instrumental assistance (physical care, symptom management, medications management) was most pronounced, but did not reach statistical significance.

**Table 2:** Unmet needs identified by respondents to the South Australian Health Omnibus in 2006, 2007 (n = 5547) who had actively provided care (day-to-day or intermittent) at some time in the five years before responding for someone at the end of life and knew whether or not they would provide care again

	**Respondents who had someone close to them die from an expected death in the 5 years before responding who died of …**					
	**…heart failure**		**…a life-limiting illness other than heart failure**			**p value**
	**n = 73**		**n = 1207**			
**Perceived additional support needed for:**	**n**	**%**	**n**	**%**		
**physical care, symptom control, medications**	24	32.9	286	23.7		0.075
**information about the disease progression of services available**	9	12.3	214	17.7		0.237
**emotional, spiritual or bereavement support**	21	28.8	297	24.6		0.424
**finances**	3	4.1	77	6.4		0.619#

# Fisher’s Exact Test used.

Age of respondent as a continuous variable, highest level education, palliative care service use, SEIFA index and the four domains of needs (Table 2) were used in the binary regression model. The model found two significant factors that helped to explain differences between caregivers for people with HF and caregivers for people with other diagnoses in unmet needs at the time of death – increasing age (OR 1.02; 95% CI 1.01 to 1.04; *p* = 0.005) and not having access to palliative care services (OR 0.39; 95% CI 0.24 to 0.64; *p* = 0.000). For this analysis, the Hosmer and Lemeshow goodness of fit (*p* = 0.665) suggested that the model adequately fits the data and the Omnibus Tests of Model coefficients (*p* = 0.000) confirmed this. The Nagelkerke R square was 0.075.

## Discussion

This study has described the characteristics of the informal HF caregiver and also identified some similarities and differences with caregivers of other conditions. The lower uptake of palliative care services by individuals with HF, compared to other conditions such as cancer, is of concern given the similar perceived needs and burden profile. This may, in practice, relate to the commonly reported challenges in determining prognosis [28]. Key findings include that in the HF population, the period of caregiving is longer than the overall mean for caregivers for people at the end of life [10].

More than 80% of active care was provided by people other than the deceased’s spouse. These data reflect, in part, the changing demographics of Australian society. Of note, the rates of caregiving provided by the next generation (sons and daughters) reflect the older age of people dying from HF. This observation also has implications for issues such as time off work and broader social and economic implications of caregiving [29]. As the burden of chronic conditions including HF, continues to increase, caregiving will continue to grow as an issue [30].

The need for information about available services and knowing what to expect, is highlighted in the person’s disease trajectory by this study and that the need for information is correlated with caregiving burden [31]. These data underscore the importance of providing patients and their families information of what to expect in the future [17].

### Study strengths

This study provides baseline data to inform the design of interventions that may better support caregivers for people with HF. First, it is the first population based study to survey a population seeking HF caregiver needs at a whole-of-population level. The sampling process employed by the Health Omnibus allows a broadly representative sample across the whole community. Second, rather than using health care providers to survey perceptions of care and comfort, the use of independent interviewers has reduced the likelihood of a biased positive appraisal of service provision. Third, the current study is a useful proxy reflection of the people’s perceptions of end of life care and is likely to relate to the deceased’s experience.

#### ***Limitations***

Interpretation of these data should be considered with the caveats of a retrospective, descriptive cross-sectional study and the limitations of a questionnaire items that was not subject to detailed comprehensive psychometric testing. This study used the reports of proxies to investigate the patient comfort, services use and place of death – the list is objective and reproducible - at the end of life. For symptom control in the terminal phases of a life-limiting illness, proxy reports are frequently used in palliative care research due to the difficulties encountered in research engaging people with a life-limiting illness [32]. Studies have examined the accuracy of retrospective reports by proxies, comparing reports of patients prior to death with those of their relatives after death [33]. In studies comparing concurrent prospective (pre-death) reports by patients and their relatives, family caregivers’ overall symptom distress scores have been highly correlated with patients’ overall scores. Families tend to rate physical symptoms more severely than patients and under-report psychological distress [34]. This is thought to occur because physical symptoms can be easier to observe than psychological distress [34]. Other factors that can affect the accuracy of proxy reports include if a caregiver is living with the person [35] and poor caregiver coping [36].

A further limitation is the *post hoc* nature of the survey precluded validation of HF status or description of the severity of HF. Any recall bias or response shift over time is likely to be distributed evenly between those who did and did not access specialist services. It is also important to note that perception of needs is related to the perception of severity, cumulative burden of symptoms and how these may change over time [37].

This study describes data from a single state, in a health system that has relatively high rates of access to specialist palliative care. The models of care and care offered differ in health settings around the world and may therefore limit the capacity to apply these findings to other health systems. There is also the potential that people may have not identified the cause of death being specifically related to HF given that caregivers’ involvement in clinical consultations may, at times, be limited.

#### ***Implications for practice or policy***

This study suggests that there is a need to consider more carefully how specialist palliative care services identify people whose needs are complex enough to warrant additional support as there are people currently not using the service who could potentially benefit from additional support. Ultimately, the responsibility of palliative care services should not be based on people who manage to navigate the health and social system in order to access current services, but a commitment by service providers to seek people with life-limiting illnesses and their caregivers whose complexity of needs warrant increased supports.

#### ***Implications for practice***

These findings have some useful implications for HF practice. Almost 30% of respondents reported an unmet need for support in providing physical care, and this is of particular importance to support community based care. In parallel with other studies there is an increased need for information about services and the disease trajectory and providing access to appropriate models of support for caregivers in the community. The method of recruitment, using population based sampling, is likely to access individuals who do are not recruited into studies in settings, such as HF clinics, where there is likely to be greater access to instrumental support.

#### ***Implications for future research***

This study has used a retrospective, cross-sectional, population-based approach which is dependent upon recollections of bereaved respondents. Future research in this area needs to validate these findings in different health systems in prospective studies. Such research would allow a better understanding of the complexities of needs at the end of life, and how best to meet these needs across the population. Rather than face-to-face interviews, computer-assisted telephone techniques or web-based technologies can potentially be used at a population level to replicate this study in other health systems. In describing the equitable access to specialist palliative care services, the needs of people with life-limiting illnesses and their caregivers need to be considered in a more comprehensive way.

Most importantly, it will be crucial to prospectively follow the perceived needs of caregivers while in the role and correlate this with long-term caregiver health outcomes [30]. Perceived caregiver needs should be explored separately to the perceptions of the person with the life-limiting illness. For caregivers, their needs continue long into the period of time following the death and how health and social services support people who years later still have ongoing grief remains a challenge.

## Conclusions

This study has contrasted similarities and differences in the caregiving experience for informal carers with HF and other conditions. As communities continue to age and less informal caregiving is available, considering the type and level of community based caregiving is likely to increase in importance. To date the majority of studies have focussed on social support [14] and the need for assistance with physical care has not been documented using a similar methodology before, and requires a focused response by service providers. These data and the work of others illustrates the complexity of the caregiving experience and the importance of assessing individuals’ needs and social circumstances.

## Competing interests

The authors have no conflicts of interest to disclose.

## Authors’ contribution

PMD: critical interpretation of the data, manuscript preparation and review; AA: study design and manuscript preparation and review; PJN critical interpretation of the data, manuscript preparation and review; KC: critical interpretation of the data, manuscript preparation and review; DCC: study design, data analysis and manuscript preparation and review. All authors read and approved the final manuscript.

## Pre-publication history

The pre-publication history for this paper can be accessed here:

http://www.biomedcentral.com/1472-6963/13/342/prepub
